# Cranial Neuropathies and Neuromuscular Weakness: A Case of Mistaken Identity

**DOI:** 10.5811/cpcem.2017.4.33728

**Published:** 2017-07-14

**Authors:** Daniel Z. Adams, Andrew King, Colin Kaide

**Affiliations:** The Ohio State University Wexner Medical Center, Department of Emergency Medicine, Columbus, Ohio

## Abstract

We describe a case of wound botulism initially thought to represent Miller-Fisher variant Guillain-Barré syndrome (MFS). Botulism classically presents with the so-called “four D’s” (diplopia, dysarthria, dysphagia, dry mouth) with symmetric, descending weakness. MFS presents with a triad of limb-ataxia, areflexia, and ophthalmoplegia, with variable cranial nerve and extremity involvement. The distinction can be difficult but is important as early initiation of botulinum antitoxin is associated with improved patient outcomes in cases of botulism. Furthermore, it is important to recognize intravenous drug use as a risk factor in the development of botulism, especially given an increase in injection drug use.

## INTRODUCTION

Botulism is a rare, neurotoxin-mediated illness produced by the gram positive, anaerobic, spore-forming bacilli *Clostridium botulinum.*^1^,^2^ For the purpose of surveillance, cases are described as transmitted through four different categories to include foodborne, wound, infant, and other. There are eight different known strains of *C. botulinum,* each with a different antigenic variant. Regardless of the means of acquisition, weakness and paralysis result when the toxin irreversibly binds with presynaptic peripheral cholinergic nerve endings (i.e. neuromuscular junction and autonomic ganglia) and prevents stimulation-induced acetylcholine release by the presynaptic nerve.^2^,^3^ Recovery results only when new nerve terminals sprout and form new synaptic contacts, a process that can take months. We present a case of wound botulism in an injection drug user that highlights the importance of vigilance in this difficult-to-diagnose illness.

## CASE REPORT

A 30-year-old male with a past medical history of type 1 diabetes presented to our emergency department (ED) with three days of worsening diplopia, dysarthria, and dysphagia. The patient stated his initial symptoms included headache and blurred vision for which he was evaluated at an outside hospital and treated for a migraine headache. Thereafter, the headache resolved, but he noted progressive diplopia with the onset of dysphagia, dysarthria, and hoarseness. The morning of his presentation to our ED he complained of inability to swallow and had difficulty handling his secretions with progressive drooling. He also noted facial weakness and difficulty ambulating. Review of systems was significant for two children at home with upper respiratory illnesses. He was otherwise well prior to his presentation.

Vital signs in the ED were normal. His physical exam was significant for a well-appearing healthy male with pooling secretions and upright posturing requiring frequent suctioning of his oropharynx. Neurologically he had significant dysarthria and hoarseness, as well as bilateral and symmetric ptosis and opthalmoplegia (cranial nerve 3 and 6 palsies), with a weak gag reflex. He had symmetrically decreased 1/4 upper extremity reflexes and 2/4 lower extremity reflexes with ataxia on ambulation. His motor and sensory exams were otherwise normal. His labs were unremarkable including a white blood cell count, serum chemistry, liver function testing, and urinalysis. Bedside negative inspiratory force was −40 cm H_2_O (normal −80 to −100 cm H_2_O), and his vital capacity was 2.8 liters (normal 3 to 5 liters). The differential diagnosis at that time included concern for Guillain-Barré syndrome (GBS), specifically Miller-Fisher syndrome (MFS), as well as neuromuscular junction disorders such as myasthenia gravis, infiltrative central nervous system processes ( i.e.lymphoma, metastatic cancer), sarcoidosis, Lyme disease, botulism and tick paralysis.

Neurology was consulted with continued monitoring in the ED. Magnetic resonance imaging of his brain was unremarkable, as were the results of lumbar puncture. Extensive laboratory testing looking for infectious or autoimmune causes were negative, including human immunodeficiency virus testing, lyme disease titers, acetylcholine receptor antibody, and a ganglioside antibody panel. He was admitted to our step-down unit for close monitoring and empirically started on intravenous immunoglubulin for MFS while awaiting other studies. Early during his admission, it was disclosed to house staff that he was an IV drug user and shared needles with multiple persons. He had withheld this information because he did not want his family to know about his drug addiction. Given his worsening inability to handle oropharyngeal secretions, he was intubated and transferred to intensive care.

An electromyography (EMG) was performed during this interval showing decreased compound muscle action potential (CMAP) amplitudes, strongly suggesting the diagnosis of botulism. Both the state health department and Centers for Disease Control and Prevention (CDC) were contacted with an immediate request for botulinum anti-toxin. Confirmatory studies for botulism were sent and empiric penicillin G was given. Botulism antitoxin was received and administered with extubation the morning after administration. Ten days after his initial presentation he was discharged well and continued to improve on outpatient follow-up.

## DISCUSSION

Wound botulism case definitions include probable and confirmed cases. A probable case is defined as “a clinically compatible case in a patient who has no suspected exposure to contaminated food and who has either a history of a fresh, contaminated wound during the 2 weeks before onset of symptoms, or a history of injection drug use within the 2 weeks before onset of symptoms.” A confirmed case is defined as “a clinically compatible case that is laboratory confirmed in a patient who has no suspected exposure to contaminated food and who has a history of a fresh, contaminated wound during the 2 weeks before onset of symptoms, or a history of injection drug use within the 2 weeks before onset of symptoms.”^4^ According to the CDC, in 2014 there were 161 confirmed and 16 probable cases of botulism reported in the United States,^5^ the vast majority of laboratory-confirmed cases were infant botulism (80%), followed by wound botulism (10%), foodborne (9%), and botulism of unknown or other etiology (1%). Of the probable cases, the majority were wound botulism cases (69%) followed by foodborne botulism (31%). Of note, a major outbreak of foodborne botulism was reported in 2015 associated with a church potluck, causing one of the largest outbreaks in U.S. history and resulting in one death. As it pertains to our case, IV drug use is a risk factor for the acquisition of botulism and something that is likely to be seen more often with the current increase in injection drug use in the U.S. 7–10

CPC-EM Capsule

What do we already know about this clinical entity?

Wound botulism is a rare clinical entity that may present with subtle initial findings. Common symptoms include diplopia, dysarthria, dysphagia, and dry mouth.

What makes this presentation of disease reportable?

Injection drug use is rising in the U.S. This paper presents a case of wound botulism that resulted from injection drug use, a rare vector of botulism.

How might this improve emergency medicine practice?

Wound botulism is a rare condition. Treatments including early administration of botulinum antitoxin and possible early mechanical ventilation can improve mortality.

How might this improve emergency medicine practice?

Given rising injection-drug abuse in the U.S, consider botulism in the differential diagnosis. Prompt identification could be life-saving.

Patients with botulism classically present with acute onset bilateral cranial nerve palsies and bulbar symptoms (the so-called “four Ds:” diplopia, dysarthria, dysphagia, and dry mouth) with a symmetric and descending flaccid weakness^11^ (Table 1). Symptoms are noticed 18–36 hours after exposure but may take days to manifest.^12^ Patients are afebrile unless there is an infected source wound. With illness onset, patients may complain of blurred vision, difficulty speaking, and difficulty swallowing as cranial nerves become involved. Physical exam will revel ptosis, extraocular muscle weakness or palsy, and a suppressed gag reflex. Pupils are typically dilated and may be unreactive to light.^12^ With illness progression, patients develop symmetric descending weakness initially involving the head and neck. Deep tendon reflexes may be diminished, and patients may have difficulty with coordination.^13^,^14^ As would be anticipated, autonomic dysfunction may lead to ileus, urinary retention, orthostatic hypotension, reduced salivation and lacrimation. The illness becomes life-threatening when respiratory muscle function is compromised and may require intubation and prolonged mechanical ventilation 1, 13, 14. While wound botulism would be expected to have an identifiable source, such as an abscess, it has been associated with simple abrasions, lacerations, open fractures, surgical incisions, and hematoma. 15

The differential diagnosis for causes of acute, life-threatening weakness and descending paralysis are listed in Table 2.^1^ Other considerations include electrolyte (i.e. hypo-hyperkalemia, hypo-hypercalcemia, hypo-hypermagnesemia, hypophosphatemia), metabolic (i.e. hypoglycemia, hyperthyroidism, hypothyroidism), endocrine (i.e. adrenal insufficiency) and toxicologic causes (i.e., organophosphate poisoning, carbon monoxide poisoning).

Our initial impression of this patient’s presentation was MFS given the predominant cranial nerve findings and minimal motor weakness. MFS is a GBS variant that classically presents with a triad of limb-ataxia, areflexia, and ophthalmoplegia with or without pupillary areflexia.^16^,^17^ Many patients, however, will not have all three classic findings. About half of patients with MFS present with facial nerve involvement with other cranial nerves variably affected. Mild sensory involvement has been reported as well. Extremity involvement in MFS is rare but can occur in about one-third of cases, as would be seen in GBS.^23^ In cases that do not progress, clinical improvement is seen in 2–4 weeks, with near complete resolution by six months. Given the similarity in symptoms between MFS and botulism, a thorough history, including recent infection, intravenous drug use, case clustering, etc., and physical examination are paramount for guiding the appropriate diagnostics and treatment. A strong recommendation can be made to specifically question patients presenting in this fashion about intravenous drug use after they have been separated from family members to facilitate truthful responses and honest communication.

Diagnosis of the various types of botulism varies, but history and physical examination are essential, as many of the diagnostic assays may be negative or take a prolonged time to result. Regarding wound botulism, attempts to isolate *C. botulinum* from a potential source wound should be attempted. Serum assays for botulinum toxin are typically negative and not helpful, and stool studies will not be helpful in wound botulism. Of patients tested in one study, only 68% of 73 patients with wound botulism had positive serum assays for botulinum toxin.^19^ EMG findings of decreased CMAP amplitudes, an incremental response to stimulation (post-activation facilitation), absence of post-activation exhaustion, and post-activation facilitation longer than two minutes are diagnostic.^20,21^

As for any cause of acute weakness in the ED, our focus should be on initial airway management, either for prevention of aspiration or impending respiratory failure. Monitoring of vital signs and respiratory parameters, such as negative inspiratory force and vital capacity, are crucial in identifying the patient’s trajectory and those that require early intubation. Thereafter, initiation of treatment for botulism should be presumptive given the diagnosis is likely to be delayed and early initiation of treatment with botulinum antitoxin might decrease mortality and reduce duration of mechanical ventilation.^22,23^ A heptavalent botulinum antitoxin is the only antitoxin available in the U.S. for patients older than one-year, with a human-derived botulism immune globulin used for infants less than one year old. The state health department and/or CDC (770-488-7100) should be contacted immediately for early acquisition and administration of heptavalent antitoxin or the California Department of Public Health Infant Botulism Treatment and Prevention Program (510-231-7600) for acquisition of BabyBIG® when appropriate.^1^

## CONCLUSION

It is increasingly important to consider botulism in the differential diagnosis of acute weakness given the increase in injection drug use seen in the U.S. A high degree of suspicion should be maintained in any person presenting with features of cranial nerve and autonomic dysfunction with or without evidence of descending weakness or paralysis. When cases are identified, early administration of botulinum antitoxin can improve outcomes. Regardless, patients may still require prolonged hospitalization and mechanical ventilation. Although a rare illness, it is important to keep botulism in the differential for patients presenting with weakness, as timely diagnosis and appropriate treatment are critical to the patient’s outcome.

## Figures and Tables

**Table 1:** Signs and symptoms of botulism.

Cranial nerves	Diplopia, dysarthria, dysphagia, dysphonia, opthalmoplegia (CN II, IV, VI), facial paralysis (CN VII), mydriasis, photophobia
Autonomic nervous system	Urinary retention/incontinence, ileus, dry mouth, orthostatic hypotension, respiratory failure
Peripheral nervous system	Descending weakness with loss of deep tendon reflexes

**Table 2:** Differential diagnosis of acute weakness.

Disease	Signs/symptoms	Diagnosis
Guillain-Barré syndrome (GBS)	Symmetric ascending weakness and paralysis with loss of deep tendon reflexes +/− autonomic dysfunction	CSF studies, EMG
Miller-Fisher variant GBS	Triad of opthalmoplegia, ataxia, and areflexia	CSF studies, EMG
Myasthenia gravis	Proximal muscle and bulbar muscle weakness with cranial nerve deficits that worsen with exertion	Ice-pack test, edrophonium stimulation test, acetylcholine receptor antibodies
Lambert-Eaton Syndrome	Proximal > distal and lower > upper extremity weakness that worsens with illness or elevated temperature and improves with repetition; may have ptosis, diplopia, and dysarthria	Voltage-gate calcium channel antibodies, search for underlying malignancy
Tick paralysis	Symmetric ascending flaccid paralysis with loss of deep tendon reflexes	Clinical diagnosis with resolution of symptoms on tick removal
